# Functional Polymorphisms in the CYP2C19 Gene Contribute to Digestive System Cancer Risk: Evidence from 11,042 Subjects

**DOI:** 10.1371/journal.pone.0066865

**Published:** 2013-07-16

**Authors:** Bo Zhou, Zhenshun Song, Mingping Qian, Liang Li, Jian Gong, Shaowu Zou

**Affiliations:** Department of General Surgery, Shanghai Tenth People's Hospital, Tongji University, Shanghai, People's Republic of China; Centro di Riferimento Oncologico, IRCCS National Cancer Institute, Italy

## Abstract

**Background:**

CYP2C19 belongs to the cytochrome P450 superfamily of enzymes involved in activating and detoxifying many carcinogens and endogenous compounds, which has attracted considerable attention as a candidate gene for digestive system cancer. CYP2C19 has two main point mutation sites (CYP2C19*2, CYP2C19*3) leading to poor metabolizer (PM) phenotype. In the past decade, the relationship between CYP2C19 polymorphism and digestive system cancer has been reported in various ethnic groups; however, these studies have yielded contradictory results.

**Methods:**

To clarify this inconsistency, we performed this meta-analysis. Databases including Pubmed, EMBASE, Web of Science and China National Knowledge Infrastructure (CNKI) were searched to find relevant studies. Odds ratios (ORs) with 95% confidence intervals (CIs) were used to assess the strength of association.

**Results:**

In total, 18 studies with 4,414 cases and 6,628 controls were included. Overall, significantly elevated digestive system cancer risk was associated CYP2C19 PM with OR of 1.66 (95%CI: 1.31–2.10, P<10^−5^) when all studies were pooled into the meta-analysis. There was strong evidence of heterogeneity (P = 0.006), which largely disappeared after stratification by cancer type. In the stratified analyses according to cancer type, ethnicity, control source and sample size, significantly increased risks were found.

**Conclusions:**

In summary, our meta-analysis suggested that the PM phenotype caused by the variation on CYP2C19 gene is associated with increased risk of digestive system cancer, especially in East Asians.

## Introduction

Cytochrome P450 2C19 (CYP2C19), one of the isoforms of CYP enzymes, is a clinically important enzyme responsible for the metabolism of a number of therapeutic drugs, such as clopidogrel, omeprazole, lansoprazole, rabeprazole, diazepam, propranolol and S-mephenytoin [1]-[6]. Besides being responsible for the metabolism of therapeutic agents, CYP2C19 is also known to be involved in the detoxification of potential carcinogen [7] or the bioactivation of some environmental procarcinogen(s) to reactive DNA binding metabolites [8]–[10]. The CYP2C19 gene locus on chromosome 10q24 [11] is currently known to encode at least twenty CYP2C19 alleles. Among them, the most important of these alleles are: CYP2C19*2 (681G>A, rs4244285) and CYP2C19*3 (636G>A, rs4986893). The nucleotide changes in the CYP2C19*2 and *3, lead to a splicing defect and stop codon, respectively, and therefore to nonfunctional proteins, hence the name poor metabolizer (PM) phenotype [12]. They represent more than 99% of all the abnormal CYP2C19 alleles in Asian population and 87% in Caucasian population. The PM phenotype has been shown to represent 13 to 23% of Asian populations, but approximately 2 to 5% of Caucasian populations [13], [14]. The wild type CYP2C19*1 gene is categorized as extensive metabolizer (EM) phenotype. These phenotypes are strongly related to metabolic capacity. In addition, phenotyping analyses revealed an association between CYP enzyme activity and the risk of developing several forms of cancer [15].

In the past decade, the polymorphic effects of two CYP2C19 genotypes/phenotypes, extensive metabolizer and poor metabolizer, on the development of digestive system cancer have been investigated among various populations. However, existing studies have yielded inconsistent results. These disparate findings may be due partly to insufficient power, false-positive results and publication biases. The interpretation of these studies has been further complicated by the use of different populations or different control source. To help clarify the inconsistent findings, we therefore conducted a comprehensive meta-analysis to quantify the overall risk of CYP2C19 polymorphisms on developing digestive system cancer as well as cancer-specific risk.

## Materials and Methods

### Literature search strategy

Epidemiological association studies published before the end of December, 2012, on digestive system cancer and CYP2C19 were sought by computer-based searches from databases including MEDLINE, PubMed, EMBASE, ISI web of science and CNKI (China National Knowledge Infrastructure) without language restriction. Search term combinations were keywords relating to the gene (“Cytochrome P450 2C19 or CYP2C19”), in combination with words related to cancer (“cancer, carcinoma, tumor or neoplasm”), and “polymorphism or variant”. The titles and abstracts of potential articles were screened to determine their relevance, and any clearly irrelevant studies were excluded. The full texts of the remaining articles were read to determine whether they contained information on the topic of interest. Furthermore, reference lists of primary studies and review articles were also reviewed by a manual search to identify additional relevant publications.

### Inclusion and exclusion criteria

Studies included in the current meta-analysis had to meet all the following criteria: (1) evaluate the association between CYP2C19 polymorphism and digestive system cancer risk; (2) case–control or cohort studies and (3) sufficient data for estimating an odds ratio (OR) with 95% confidence interval (CI). The major reasons for exclusion of studies were as follows: (1) overlapping data; (2) case-only studies; (3) family based studies and review articles.

### Data extraction

Information was carefully extracted from all eligible publications independently by two of the authors according to the inclusion criteria listed above. For each study, the following characteristics were collected: first author, publication year, ethnicity, cancer type, number of cases and controls, age, sex, source of control groups (population-based controls or hospital-based controls), Hardy–Weinberg equilibrium (HWE) status among controls, genotype/phenotype frequency in cases and controls, and genotyping methods. Discrepancies in data extraction were resolved by discussion between all authors through consensus.

### Statistical methods

For the CYP2C19 gene, we estimated the risks of PM genotype/phenotype compared with EM genotype/phenotype status between cancer patients and controls. The strength of association between the CYP2C19 gene and cancer risk was assessed by OR with the corresponding 95% CI. Heterogeneity was evaluated with standard chi-square-based Q test [16]. Both fixed-effects (Mantel–Haenszel method) [17] and random-effects (DerSimonian–Laird method) [18] models were performed to calculate the pooled ORs. Owing to a priori assumptions about the likelihood of heterogeneity between primary studies, the random-effects model, which usually is more conservative, was chosen. Ethnic group, cancer types, source of controls, the study size (No. of cases <300 and ≥300), were prespecified as characteristics for assessment of heterogeneity. Ethnic group was defined as Caucasian (i.e., people of European origin), East Asian (e.g., Chinese, Japanese), and other ethnic populations. Sensitivity analysis was performed by analyzing the influence of each study on the overall estimates and heterogeneity. Funnel plots and Egger's linear regression test were used to assess evidence for potential publication bias [19]. The analysis was conducted using the STATA software (version 10.0; Stata Corporation, College Station, TX). The type I error rate was set at 0.05. All the P-values were for two-sided analysis.

## Results

### Characteristics of studies

A total of 18 studies with 4414 digestive system cancer cases and 6628 controls were retrieved based on the search criteria for digestive system cancer susceptibility related to the CYP2C19 polymorphisms (Figure S1). The main study characteristics were summarized in Table 1 [20]–[36]. These studies indicate that the distribution of genotypes in controls was consistent with HWE. All of the cases were pathologically or histologically confirmed.

**Table 1:** Characteristics of the studies included in the meta-analysis.

Study	Year	Ethnicity	Case type	Control source	No. of cases/controls	Genotyping method
Shi [20]	2012	Chinese	Esophagus cancer	Population	350/350	PCR-RFLP
Sainz [21]	2011	German	Colorectal cancer	Population	1759/1776	SNPlex
Isomura [22]	2010	Japanese	Biliary tract cancer	Hospital	65/566	PCR-RFLP
Chang [23]	2010	Chinese	Hepatocellular carcinoma	Population	68/254	AS-PCR
Hu [24]	2010	Chinese	Colorectal cancer	Hospital	117/109	PCR-RFLP
Zhang [25]	2009	Chinese	Esophagus cancer	Hospital	46/38	PCR-RFLP
Yang [26]	2008	Chinese	Colorectal cancer	Hospital	83/112	PCR-RFLP
Jiang [27]	2008	Chinese	Hepatocellular carcinoma	Hospital	48/88	PCR-RFLP
Zhou [28]	2006	Chinese	Esophagus cancer	Hospital	127/254	PCR-RFLP
Tamer [29]	2006	Turkish	Colorectal cancer, Gastric cancer	Hospital	105, 77/105	RT-PCR
Mochizuki [30]	2005	Japanese	Hepatocellular carcinoma	Population	44/843	PCR-RFLP
Sugimoto [31]	2005	Japanese	Gastric cancer	Hospital	111/315	PCR-RFLP
Landi [32]	2005	Spanish	Colorectal cancer	Hospital	351/321	APEX
Shi [33]	2004	Chinese	Esophagus cancer, Gastric cancer	Population	135, 148/372	PCR-RFLP
Sachse [34]	2002	British	Colorectal cancer	Population	490/592	PCR-RFLP
Chau [35]	2000	Japanese	Hepatocellular carcinoma	Population	29/186	PCR-RFLP
Tsuneoka [36]	1996	Japanese	Hepatocellular carcinoma	Hospital	16/64	PCR-RFLP
Unpublished data	/	Chinese	Colorectal cancer, Hepatocellular carcinoma	Hospital	125, 120/283	PCR-RFLP

### Quantitative Data Synthesis

**Figure 1:**
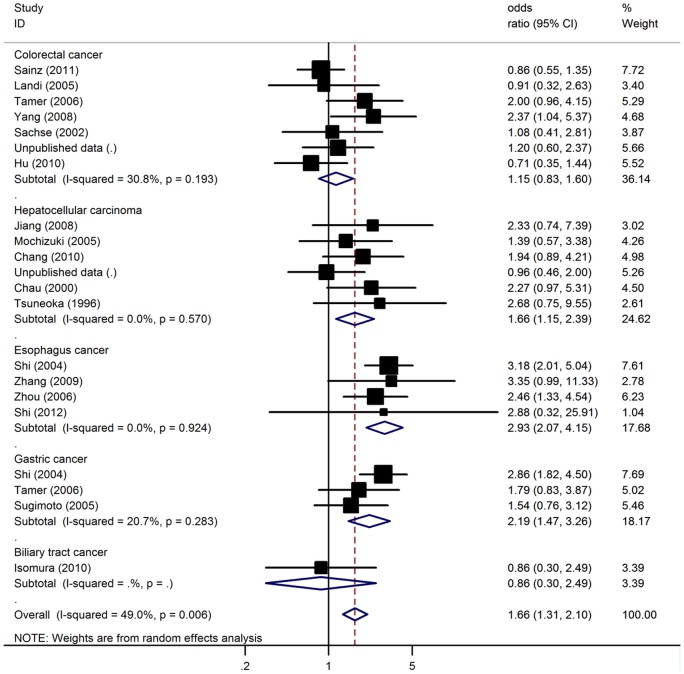
Meta-analysis with a random-effects model for the association between digestive system cancer risk and CYP2C19 PM genotype.

Using random effect model, the overall OR of the PM genotype for digestive system cancer was 1.66 [95% CI: 1.31–2.10, P(Z)<10^− 5^, P(Q) = 0.006; Figure 1]. In subgroup analyses by cancer types, significantly increased cancer risks were found for gastric cancer (OR = 2.19, 95%CI: 1.47–3.26, P<10^−4^), esophagus cancer (OR = 2.93, 95%CI: 2.07–4.15, P<10^−5^) as well as hepatocellular carcinoma (OR = 1.66, 95%CI: 1.15–2.39, P = 0.006). Unfortunately, no significant association was found for colorectal cancer and biliary tract cancer. When studies were stratified for ethnicity, significant risk was found among East Asians with OR of 1.84 (95%CI: 1.42–2.38, P<10^−5^). However, no significant associations were detected among Caucasian and other ethnic populations (Table 2). Subsidiary analyses of controls source yielded an OR for hospital based controls of 1.50 (95% CI: 1.14–1.96, P = 0.003) and for population based controls of 1.88 (95% CI: 1.25–2.83, P = 0.002). In considering sample size subgroups, the OR was 1.85 (95% CI: 1.46–2.33, P<10^−5^) in small studies compared to 0.93 (95% CI: 0.64–1.35, P = 0.71) in large studies. Significant between-study heterogeneity was detected in the overall analysis (P = 0.006). However, the heterogeneity decreased sharply when studies were stratified by types of cancer.

**Table 2:** Main results of overall and subgroups in the meta-analysis.

Sub-group analysis	No. of datasets	OR (95%CI)	P(Z)	P(Q)
Overall	21	1.66 (1.31–2.10)	<10^−5^	0.006
Cancer type				
Colorectal cancer	7	1.15 (0.83–1.60)	0.40	0.19
Gastric cancer	3	2.19 (1.47–3.26)	<10^−4^	0.28
Esophagus cancer	4	2.93 (2.07–4.15)	<10^−5^	0.92
Hepatocellular carcinoma	6	1.66 (1.15–2.39)	0.006	0.57
Biliary tract cancer	1	0.86 (0.30–2.49)	0.78	NA
Ethnicity				
East Asian	16	1.84 (1.42–2.38)	<10^−5^	0.04
Caucasian	3	0.90 (0.61–1.32)	0.58	0.92
Others	2	1.90 (1.12–3.22)	0.02	0.84
Control source				
Poulation	9	1.88 (1.25–2.83)	0.002	0.005
Hospital	12	1.50 (1.14–1.96)	0.003	0.19
Study size				
Cases <300	17	1.85 (1.46–2.33)	<10^−5^	0.06
Cases ≥300	4	0.93 (0.64–1.35)	0.71	0.75

NA: not available.

### Sensitivity analyses and Publication bias

A single study involved in the meta-analysis was deleted each time to reflect the influence of the individual dataset to the pooled ORs, and the corresponding pooled ORs were not qualitatively altered. Begg's funnel plot and Egger's test were performed to access the publication bias of the literatures. The shape of the funnel plots was symmetrical (Figure S2). The statistical results still did not show publication bias in these studies (Egger's test: *P* = 0.73, Figure S3).

## Discussion

Large sample and unbiased epidemiological studies of predisposition gene polymorphisms could provide insight into the in vivo relationship between candidate genes and complex diseases. The present meta-analysis provides the first comprehensive assessment of the risk of digestive system cancer and CYP2C19 gene polymorphisms. Its strength was based on the accumulation of published data giving greater information to detect significant differences. In total, the meta-analysis involved 17 studies for digestive system cancer which provided 4414 cancer cases and 6628 controls. Our results demonstrated that the PM genotype of CYP2C19 is a risk factor for developing digestive system cancer. However, this association became non-significant when the meta-analysis was restricted to larger studies, suggesting a potential small studies effect. Data from the studies did not exhibit statistically significant heterogeneity in the majority of contrasts.

We found that CYP2C19 PM genotype, in stratified analysis by cancer type, was statistically related with elevated risks for gastric cancer, esophagus cancer and hepatocellular carcinoma. However, we did not observe any significant association between the genetic variant and the susceptibility of colorectal cancer and biliary tract cancer. There are some possibilities for this discrepancy among tumor sites. Firstly, the tissue specificity leads to different cancer susceptibilities in different tissues. Secondly, the relative small amount of eligible studies in stratified analysis might induce significant/insignificant association by chance due to insufficient statistical power [37].

In the subgroup of ethnicity, we found significant association between CYP2C19 genotype and increased risks of digestive system cancers in Asians but not in Caucasians. Inconsistency between the two ethnicities can be explained by the possibility that different ethnic groups live with multiple life styles and environmental factors and thus yield diverse gene-environment interactions [38]. And different populations carry different genotype and/or allele frequencies of this locus polymorphism and may lead to various degrees of cancer susceptibility [39]. Relative small sample size in Caucasians might cause the inconspicuousness also.

A number of factors predict digestive system cancer, however, detailed pathogenesis mechanisms remain a matter of speculation. CYP2C19-one of the most important cytochrome P450s, is known as a key enzyme in the in vivo metabolism of a number of carcinogens and endogenous compounds, as well many structurally unrelated drugs such as omeprazole, lansoprazole, progunil, mephenytoin and citalopram. Individuals can be divided into two groups, poor metabolizers (PMs) and extensive metabolizers (EMs), depending on the hydroxylation ability of S-mephenytoin. There are two main enzyme deficient alleles called CYP2C19*2 and CYP2C19*3. An individual who inherits two mutant CYP2C19 alleles, whatever same kind (*2/*2, *3/*3) or different kind (*2/*3), has a reduced capacity to metabolize CYP2C19 substrates and is a PM. Individuals who are homozygous (*1/*1) or heterozygous (*1/*2, *1/*3) for wild-type CYP2C19*1 have efficient enzyme to metabolize CYP2C19 substrates and are EMs. Although there are several other reports about rare enzyme defect alleles, it is recognized that the purpose of prediction of CYP2C19 phenotype can be achieved by genotyping CYP2C19 only with CYP2C19*2 and CYP2C19*3 in Chinese population [40]. In addition, investigations revealed that CYP2C19 genotypes were in complete accordance with phenotypes in Japanese subjects [41], [42]. Furthermore, genotyping was applied for a mutation that correctly predicts the CYP2C19 PM phenotype in more than 90% of Caucasians [43]. These studies enabled us to predict the PM and EM phenotypes from CYP2C19 genotyping results and the PM genotypes may be more liable to metabolically activate mutagens and carcinogens.

Meta-analysis is a retrospective research that is subject to the methodological deficiencies of the included studies and several specific details merit consideration in the current meta-analysis. A first consideration is that our results are based on unadjusted estimates and a more precise analysis stratified by age, different lifestyle related habits and different grades of digestive system cancer could be performed if individual data were available. A second consideration is that the subgroup meta-analyses on ethnic populations, cancer types and source of controls are based on a small number of studies with such information available. Nevertheless, the total number of subjects included in this part of the analysis comprises the largest sample size so far. Finally, as with any meta-analysis of published results, the quality of our meta-analysis depends on that of individual studies. Ideally we would like to pool individual-level data. However this is not possible for the present study. These considerations may distort our results.

To conclude, our meta-analysis demonstrated an association between CYP2C19 PM genotype and digestive system cancer risk among Asians, but not among Caucasians. Nevertheless, large-scale and well-designed studies are needed to investigate gene–gene and gene–environment interactions on these two polymorphisms and cancer risk, which may eventually lead to better comprehensive understanding of the possible roles in tumorigenesis.

This meta-analysis is guided by the PRISMA statement (Checklist S1).

## Supporting Information

Figure S1 **Flow chart indicates the inclusion and exclusion of studies.**
(TIF)

Figure S2 **Begg's funnel plot of CYP2C19 PM genotype and digestive system cancer.**
(TIF)

Figure S3 **Egger test of CYP2C19 PM genotype and digestive system cancer.**
(TIF)

Checklist S1 (DOC)
