# Supplementary figures and images for: Functional Polymorphisms in the CYP2C19 Gene Contribute to Digestive System Cancer Risk: Evidence from 11,042 Subjects
Source: PLoS One. 2013 Jul 16;8(7):e66865. doi: 10.1371/journal.pone.0066865 (PMC3712993; doi:10.1371/journal.pone.0066865)

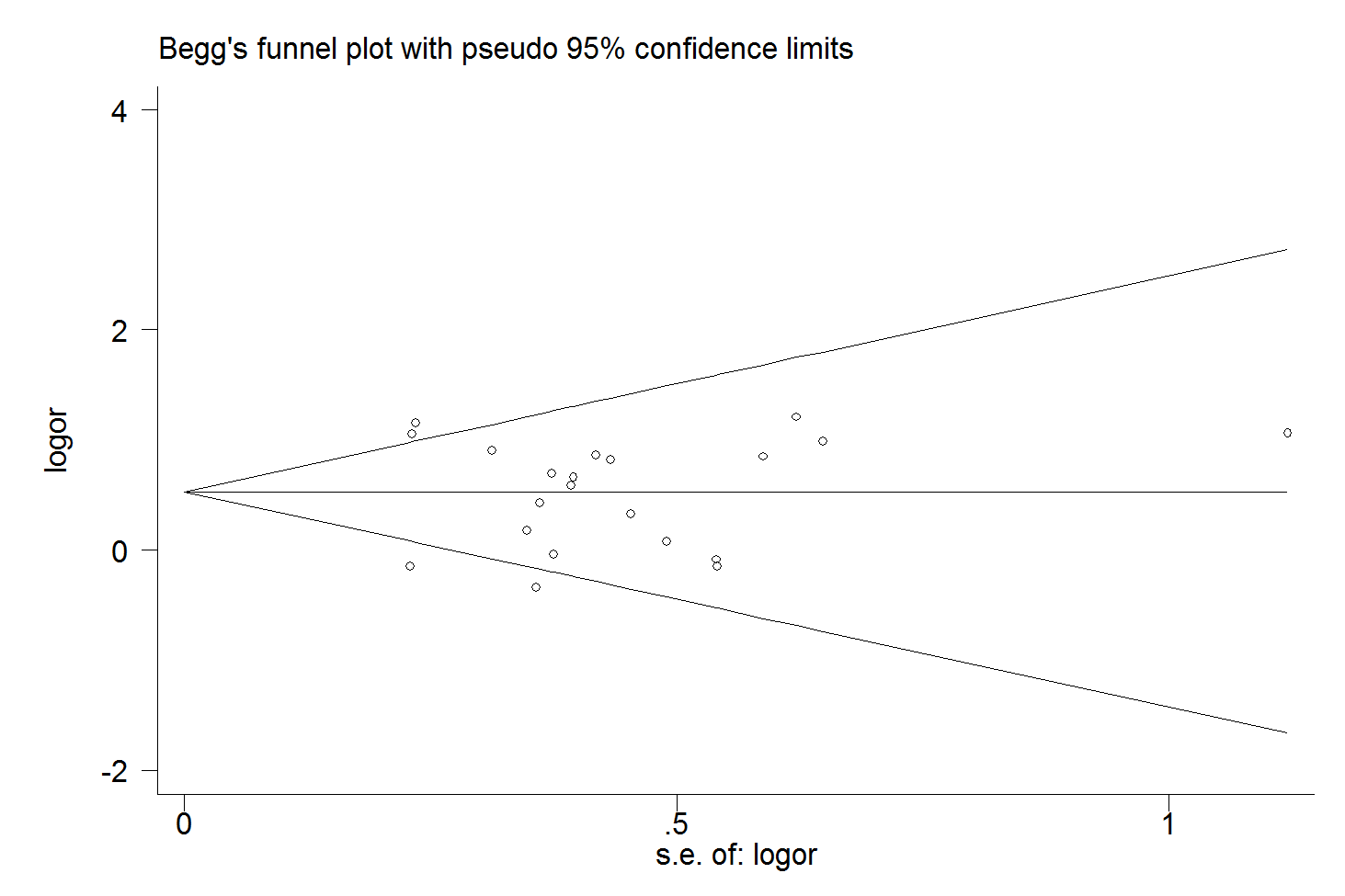

Supplement: Figure S2 — Begg's funnel plot of CYP2C19 PM genotype and digestive system cancer. (TIF) [file pone.0066865.s002.tif]

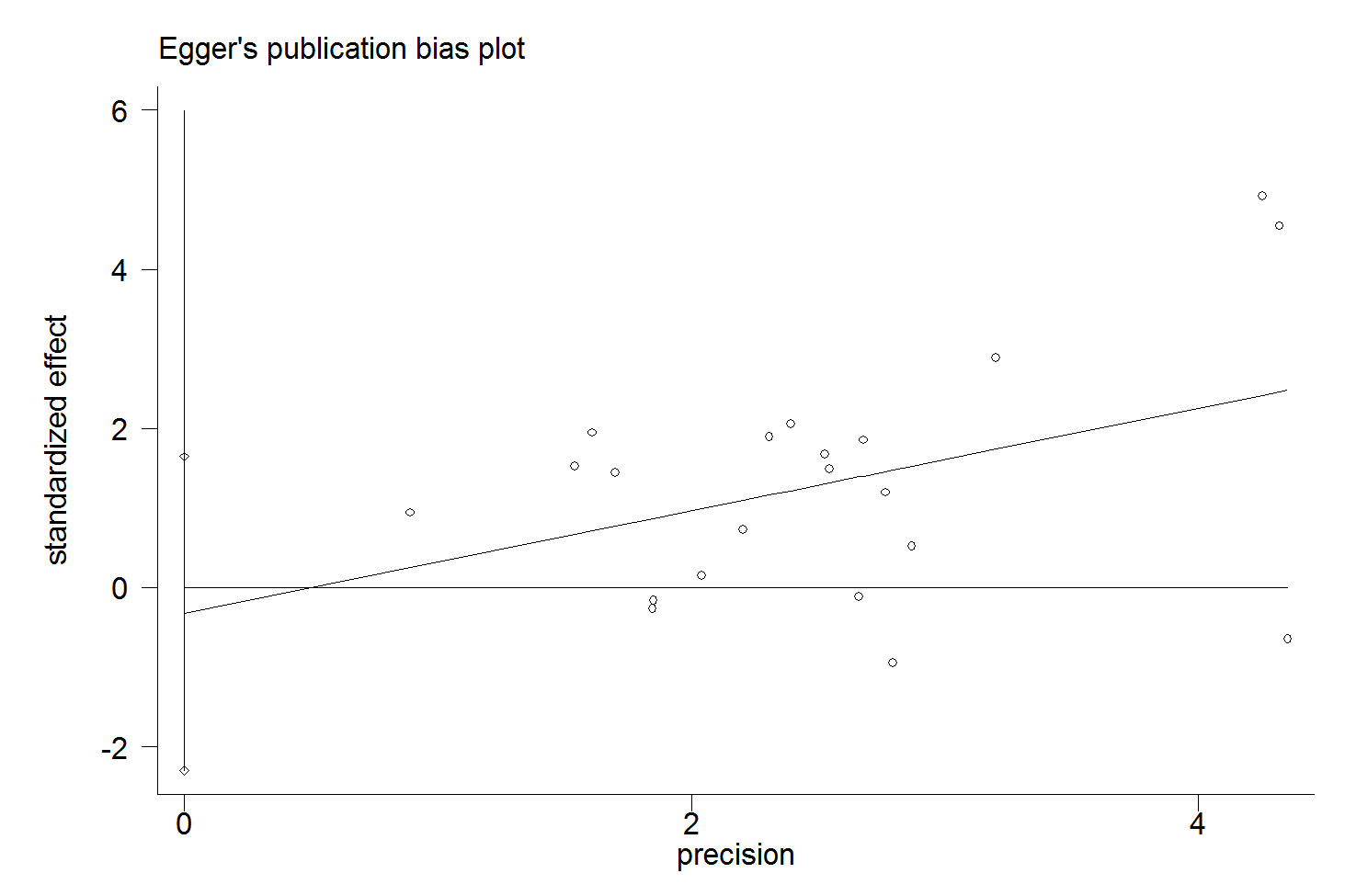

Supplement: Figure S3 — Egger test of CYP2C19 PM genotype and digestive system cancer. (TIF) [file pone.0066865.s003.tif]
